# EMG patterns during assisted walking in the exoskeleton

**DOI:** 10.3389/fnhum.2014.00423

**Published:** 2014-06-16

**Authors:** Francesca Sylos-Labini, Valentina La Scaleia, Andrea d'Avella, Iolanda Pisotta, Federica Tamburella, Giorgio Scivoletto, Marco Molinari, Shiqian Wang, Letian Wang, Edwin van Asseldonk, Herman van der Kooij, Thomas Hoellinger, Guy Cheron, Freygardur Thorsteinsson, Michel Ilzkovitz, Jeremi Gancet, Ralf Hauffe, Frank Zanov, Francesco Lacquaniti, Yuri P. Ivanenko

**Affiliations:** ^1^Laboratory of Neuromotor Physiology, Santa Lucia FoundationRome, Italy; ^2^Centre of Space Bio-medicine, University of Rome Tor VergataRome, Italy; ^3^Spinal Cord Rehab Unit and CaRMA Lab, Santa Lucia FoundationRome, Italy; ^4^Biomechanical Engineering, Delft University of TechnologyDelft, Netherlands; ^5^Biomechanical Engineering, University of TwenteEnschede, Netherlands; ^6^Laboratory of Neurophysiology and Movement Biomechanics, Université Libre de BruxellesBrussels, Belgium; ^7^OSSURReykjavík, Iceland; ^8^Space Applications Services N.V./S.A.Zaventem, Belgium; ^9^ANT NeuroBerlin, Germany; ^10^Department of Systems Medicine, University of Rome Tor VergataRome, Italy

**Keywords:** robotic exoskeleton, assisted gait, EMG patterns, spinal cord injury, neuroprosthetic technology

## Abstract

Neuroprosthetic technology and robotic exoskeletons are being developed to facilitate stepping, reduce muscle efforts, and promote motor recovery. Nevertheless, the guidance forces of an exoskeleton may influence the sensory inputs, sensorimotor interactions and resulting muscle activity patterns during stepping. The aim of this study was to report the muscle activation patterns in a sample of intact and injured subjects while walking with a robotic exoskeleton and, in particular, to quantify the level of muscle activity during assisted gait. We recorded electromyographic (EMG) activity of different leg and arm muscles during overground walking in an exoskeleton in six healthy individuals and four spinal cord injury (SCI) participants. In SCI patients, EMG activity of the upper limb muscles was augmented while activation of leg muscles was typically small. Contrary to our expectations, however, in neurologically intact subjects, EMG activity of leg muscles was similar or even larger during exoskeleton-assisted walking compared to normal overground walking. In addition, significant variations in the EMG waveforms were found across different walking conditions. The most variable pattern was observed in the hamstring muscles. Overall, the results are consistent with a non-linear reorganization of the locomotor output when using the robotic stepping devices. The findings may contribute to our understanding of human-machine interactions and adaptation of locomotor activity patterns.

## Introduction

Exoskeleton robotic devices are now often used in the rehabilitation practice to assist physical therapy of individuals with neurological disorders (Sale et al., 2012; Moreno et al., 2013). To provide patients with some degree of locomotion capability, passive (unpowered) orthoses are often prescribed (Hsu et al., 2008). However, passive devices have many limitations, including the high energy expenditure and low utilization by individuals with severe walking impairments (Wang et al., submitted). Active (powered) exoskeletons and new control implementations are extensively developed in recent years to provide new possibilities for severely paralyzed patients to walk (Fitzsimmons et al., 2009; Swinnen et al., 2010; Cheron et al., 2012; del-Ama et al., 2012; Roy et al., 2012; Sale et al., 2012; Wang et al., submitted). Many of these devices include some form of body weight support and adjustable levels of robotic guidance forces.

Investigating locomotor responses in individuals after neurological lesions, as well as in healthy subjects, when using the robotic devices, is fundamental to the development of improved rehabilitation strategies and to explore the mechanisms involved in improving locomotor function (Ivanenko et al., 2013). Even in neurologically intact subjects, the use of external devices for stepping can affect motor patterns (Hidler and Wall, 2005; Lam et al., 2008; Van Asseldonk et al., 2008; Moreno et al., 2013), modify the “locomotor body scheme” and result in distortions in the body and space representation (Ivanenko et al., 2011). There is still a lack of knowledge on the effect of robotic gait assistance on the locomotor function and its recovery in injured humans due to the complex nature of the control of locomotion, compensatory strategies, and plasticity of neuronal networks.

Several studies emphasized the importance of minimizing passive guidance and stabilization provided during gait rehabilitation (Israel et al., 2006), establishing baseline patterns (Hidler and Wall, 2005) and reduction of metabolic cost of ambulant exoskeletons (Malcolm et al., 2013). Different artificial control schemes can induce different locomotor patterns. Here we used a control strategy of the exoskeleton consisting in weight shift to the stance side to trigger a step and to provide predefined reference joint trajectories (Wang et al., 2013). This exoskeleton assisted both posture (knee stabilization during stance, weight shift, lateral stabilization) and leg movements. The main purpose of this study was to report the muscle activation patterns in a sample of intact and injured subjects while walking with a robotic exoskeleton and, in particular, to quantify the level of muscle activity during assisted gait. It can be argued that robotic-guided walking should reduce leg muscle activity in healthy subjects to a lower level and might affect the motor output in patients as well. To verify this hypothesis, we investigated the adaptation of muscle activation patterns in neurologically intact human adults and spinal cord injury (SCI) patients using a recently developed exoskeleton (called MINDWALKER, https://www.mindwalker-project.eu).

## Methods

### Participants

Six healthy volunteers (age range between 21 and 36 years, five males and one female, mean height 1.72 ± 0.09 m [mean ±*SD* (standard deviation)], weight 69 ± 12 kg) participated in this study. We also tested the MINDWALKER exoskeleton on SCI subjects (Table 1). Patient inclusion criteria were the following: age 18–45 years, traumatic/non-traumatic SCI, at least 5 month after injury with stable neurological score, complete lesion (AIS A, B at the time of inclusion) from below T7, inability to ambulate over ground without at least moderate assistance, Mini-Mental State Examination score >26. Exclusion criteria were: presence of transmissible diseases, such as (but not limited to) hepatitis, human immunodeficiency virus or Creutzfeldt-Jacob disease, symptomatic orthostatic hypotension or 30-mmHg drop when upright, subjects with spine-stabilizing devices for whom their treating surgeon contraindicates gait, contraindications for lower extremities weight bearing (pelvic or leg fracture, chronic joint pain), untreatable chronic pain, untreatable spasticity (Ashworth scale score >3), severe reduction in lower limb joint's range of motion, pressure sore stage 2 or higher, skin injuries or problems such as blisters, burns, wounds from operation, or other superficial wounds at the scalp, debilitating disease prior to SCI that causes exercise intolerance and limits mobility-related self-care and instrumental activities of daily living, premorbid major depression or psychosis, suicide attempt caused the SCI, unlikely to complete the intervention or return for follow-up, participation in another research. The studies conformed to the Declaration of Helsinki, and informed consent was obtained from all participants according to the procedures of the Ethics Committee of the Santa Lucia Foundation.

**Table 1:** **Subject characteristics**.

**Patient**	**Age, year**	**Gender**	**Weight, kg**	**Height, m**	**Lesion level**	**ASIA**	**Aethiology**	**Lesion time, months**
p1	19	M	50	1.80	T12-L1	B	Trauma	5
p2	21	M	67	1.78	T7	A	Trauma	26
p3	22	M	70	1.80	T11-T12	A	Trauma	36
p4	43	M	78	1.74	T9-T10	A	Trauma	49

Lesion level indicates the clinical neurological level, lesion time the time interval between lesion diagnosis and data recording.

### Brief description of mindwalker exoskeleton

**Figure 1:**
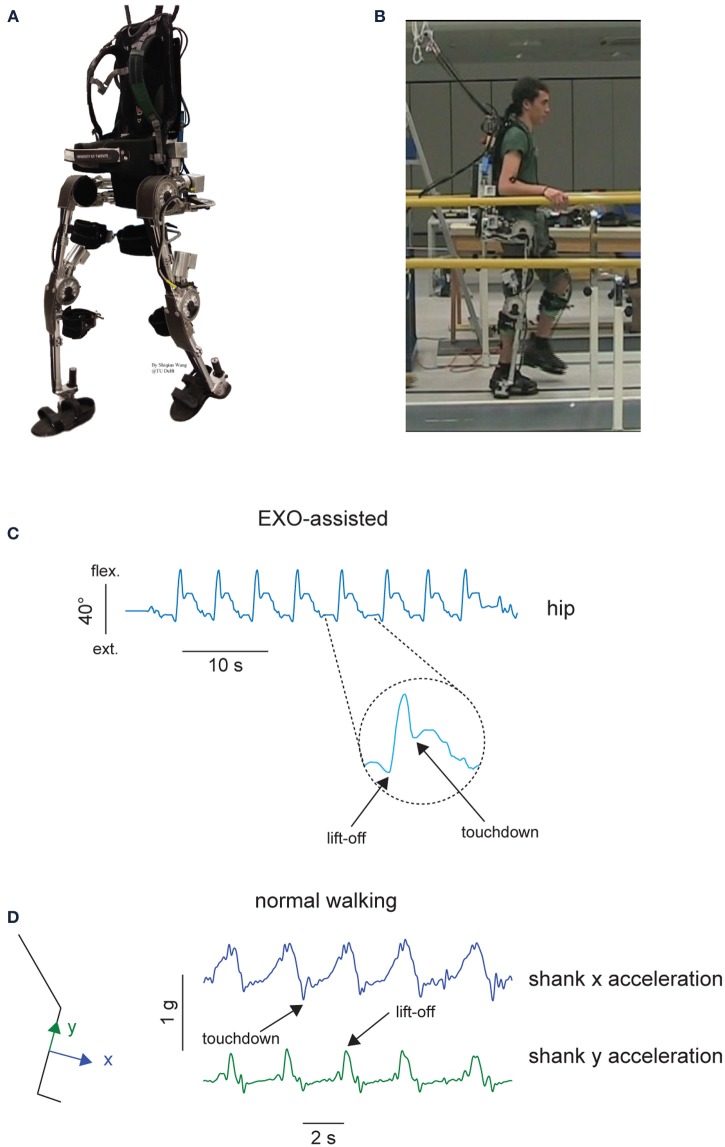
**Experimental setup. (A)** MINDWALKER exoskeleton. Each leg has five degrees of freedom. Shank and thigh segments have telescopic tubular structure to accommodate different subject statues. The exoskeleton is attached to the wearer at five main locations: footplate, shank, thigh, pelvis, and torso. Footplates are made of carbon fiber and have braces to host human feet. Shank braces are used to support most of the weight of the user in standing and walking while thigh braces are added to loosely constrain the upper leg and support the wearer during standing up. Pelvis and backpack braces are used to attach the upper body to the wearer. **(B)** A healthy subject during walking in the exoskeleton. **(C)** Definition of touchdown and lift-off events from the hip joint angle during walking in the exoskeleton. **(D)** Definition of touchdown and lift-off events from the shank inertial sensor accelerations during normal walking.

The detailed description of the exoskeleton and its control is provided elsewhere (Wang et al., 2013; Wang et al., submitted). Briefly, this exoskeleton is aimed at providing a research prototype that can empower lower limb disabled people (especially SCI patients) to walk on level ground (Figure 1A). Based on human anatomy and joint range of motion (RoM), the desired degrees of freedom (five at each leg) and joint RoM for the exoskeleton are specified to allow sitting, standing, and walking. In each leg, three degrees of freedom (DoFs: hip ab/adduction, hip flexion/extension and knee flexion/extension) are powered by series elastic actuators and two DoFs (hip endo/exo rotation and ankle dorsi/plantar flexion), are passively sprung with certain stiffness (800 and 180 Nm/rad, respectively). The exoskeleton weighs 28 kg excluding batteries and it bears its own weight by transferring the weight via its footplates to the ground. The exoskeleton can be attached to the wearer at five main locations: footplate, shank, thigh, pelvis, and torso (Figure 1A).

A finite-state machine based controller was implemented for providing gait assistance in both sagittal and frontal planes and the swing phase initiation was triggered using trunk motion (Wang et al., 2013). For example, leaning to the left and forward triggers a right step: when the estimated center of mass (CoM) falls into a predefined region, the controller detected the intention of the subject and initiates assisted weight shift to left. Then the state transits automatically to right swing. Weight shift is initiated by the subject and completed by the exoskeleton. This control strategy is relatively simple, as well as it takes advantage of natural lateral trunk oscillations that always accompany normal walking (Cappellini et al., 2010).

**Figure 2:**
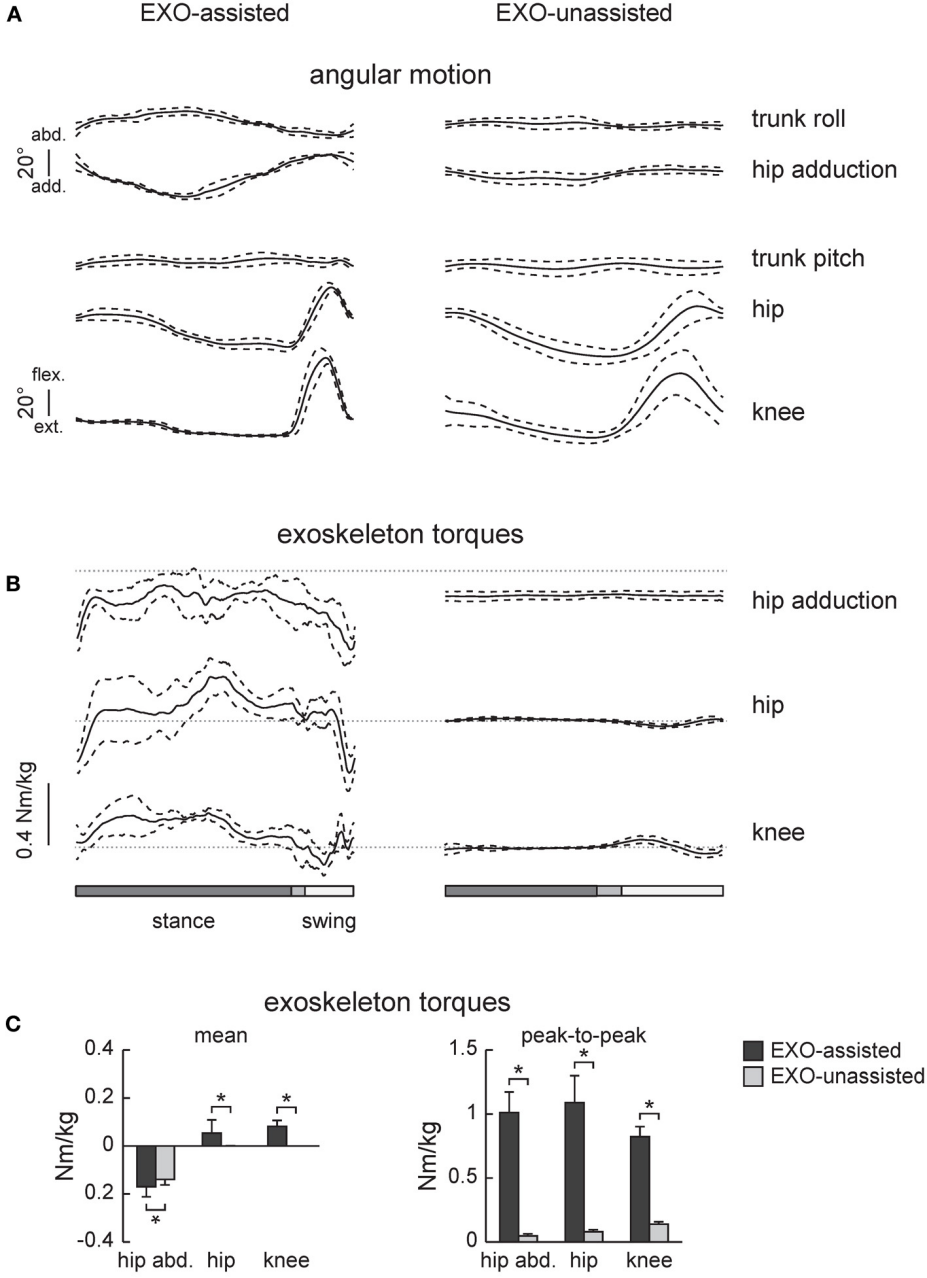
**Joint angles and exoskeleton torques recorded in healthy subjects during walking in the exoskeleton (EXO-assisted and EXO-unassisted). (A)** Ensemble-averaged (mean ±*SD*, *n* = 6) joint angular movements. **(B)** Ensemble-averaged joint torques recorded in three powered actuators of the exoskeleton (knee and hip flexion/extension and hip ab/adduction). Note little torques in the hip and knee joints in the zero-torque mode (right panel) due to the absence of assistance. **(C)** Mean torques and peak-to-peak oscillations of torques. Asterisks denote significant differences.

In this study, two control modes of the exoskeleton were used, namely, “EXO assisted” and “EXO-unassisted.” In the EXO-unassisted mode, healthy subjects wore the exoskeleton with all motorized joints in torque control mode, in which the references were 0 torque. In this mode, the exoskeleton joints were moved by the human. As the controller bandwidth is limited (Wang et al., 2013) the actual exerted torques by the exoskeleton will not be zero and therefore we quantified the actual torques measured by exoskeleton sensors (see Results and Figure 2B). In the EXO-assisted mode, all exoskeleton joints were following predefined joint angles provided with variable joint impedances, the walking trajectories during the swing phase (reference joint angles) were defined based on walking patterns of a healthy subject walking in the MINDWALKER exoskeleton in the EXO-unassisted mode (Wang et al., 2013). Hip and knee flexion angles were slightly increased during swing to ensure sufficient foot clearance. Since the reference trajectories for the swing phases were predefined, the swing phase durations were similar across conditions.

### Experiment description

Four experimental conditions in healthy individuals were recorded in the same experimental session: EXO-assisted, EXO-unassisted, NM slow, NM self-selected. “NM slow” referred to normal slow walking without the exoskeleton and “NM self-selected” normal walking at self-selected speed without the exoskeleton. In the first two conditions, the participants were asked to walk along a 8-m walkway and were allowed to place the abducted arms on horizontal handrails located at the side of the walkway (Figure 1B), to provide stability/assistance if needed. A safety harness worn by the wearer was attached to an overhead suspension system moving along with the wearer, which only came into action when the subject fell. Typically, we collected the data from 2–4 trials while walking in the exoskeleton following a short period of training (1–2 trials). On average, 8–15 strides were recorded and analyzed in each experimental condition. The total duration of the experimental session was about 1–2 h. In the EXO-assisted condition, the subjects were instructed to move their CoM forward and toward a side to trigger a contralateral step (for example leaning to the left and forward would trigger a right step). In the EXO-unassisted condition, the subjects were told to just walk at their preferred pace bearing the exoskeleton to reach the end of the walkway.

In the latter two conditions, healthy subjects walked without the exoskeleton along a 8-m walkway at slow and self-selected speeds. Gait initiation and gait termination strides were excluded from the analysis. About 10 strides were analyzed in each subject in each condition. The high speeds of normal walking were not recorded because walking in the exoskeleton was rather slow (see Results).

One experimental condition in SCI participants was recorded, that is the EXO-assisted condition. A similar protocol as used in healthy individuals (control) was employed, for comparison. In participants with complete lesions, familiarization with MINDWALKER usage was more difficult and required several days of exoskeleton training (2 or 3 times/week) for a total of session ranging between 5 and 8. SCI participants achieved the control of balance holding the handrails located at the side of the walkway. All subjects presented high motivation since the first trial and throughout testing and the comparison between the first and last sessions for the whole group of patients present only minimal changes in the mean walking speed. No clinical changes were observed, between first and last trials, in the clinical scales (the detailed description of behavioral assessment and the physiological cost index are provided elsewhere, Pisotta et al., submitted), indicating that MINDWALKER usage does not affect the functional neurological status, consistent with a limited effectiveness of robot-assisted gait training in severely paralyzed individuals (Swinnen et al., 2010; Roy et al., 2012; Sale et al., 2012). Here we analyzed the stepping pattern in the last session, after familiarization with MINDWALKER usage.

### Data recording, processing, and gait event detection

In the exoskeleton walking conditions (EXO-assisted and EXO-unassisted), joint angles and torques at aforementioned powered DoFs were recorded by the MINDWALKER exoskeleton at 1000 Hz and downsampled at 100 Hz to be used with the muscle activity recordings (Wang et al., 2013). Gait cycle events (touchdown and lift-off) were defined based on the kinematic data of the hip flexion-extension angle: touchdown as the first local minimum following the maximum and lift-off as the first local minimum preceding the maximum (Figure 1C). These kinematic criteria were verified by comparison with the events detected by inertial signals from the sensor placed on the TA muscle using a similar method as during normal walking (Jasiewicz et al., 2006). In general, the difference between the time events measured from kinematics (Figure 1C) and inertial sensors was less than 4%. We divided the recorded kinematic and kinetic data into gait cycles (touchdown as the beginning of the gait cycle), then interpolated each stride to 200 time points, and finally averaged across gait cycles (individually for each subject). Joint torques were normalized to the total body weight (subject + exoskeleton) prior to averaging across subjects. It is worth noting that these are not the net joint torques of the subjects but the resulting torques exerted by the exoskeleton to move the subject's limbs and whole body.

Electromyographic (EMG) activity was recorded by means of surface electrodes from 11 muscles simultaneously on the right side of each subject. These included vastus medialis (VM), rectus femoris (RF), biceps femoris long head (BF), semitendinosus (ST), tibialis anterior (TA), medial gastrocnemius (MG), and soleus (Sol), anterior deltoid (DELTa), posterior deltoid (DELTp), flexor carpi ulnaris (FCU), extensor carpi ulnaris (ECU). We placed EMG electrodes based on suggestions from SENIAM (seniam.org), the European project on surface EMG, and by palpating to locate the muscle bellies and orienting the electrodes along the main direction of the fibers (Winter, 1991; Kendall et al., 2005). All EMGs were recorded at 2000 Hz using a Delsys Trigno Wireless System (Boston, MA).

The EMG sensors of the Delsys Trigno Wireless System also contained 3D accelerometers, and we recorded and filtered (5 Hz low-pass zero-lag 4th order Butterworth) these inertial signals from the sensor placed on the TA muscle in order to define the gait cycle during walking without the exoskeleton (NM slow and NM self-selected walking): based on the method of Jasiewicz et al. (2006), touchdown was identified by minima in the shank x acceleration while lift-off was identified by maxima in the shank y acceleration (Figure 1D).

### EMG data analysis

EMG data were processed using standard filtering and rectifying methods. We applied a 60 Hz high-pass filter, then rectified the EMG signals and applied a 10 Hz low-pass filter (all filters, zero-lag 4th order Butterworth). EMG data were time-interpolated over a time base with 200 points for individual gait cycles (*i* = 1 ÷ 200) and averaged.

In addition to computing the ensemble-averaged EMG waveforms (Winter, 1991; Perry, 1992), we calculated for each muscle and each subject the mean and maximum EMG activity and the center-of-activity (CoA) throughout the gait cycle. The CoA during the gait cycle was calculated using circular statistics (“circ_mean.m” function in the CircStat Matlab toolbox, Berens, 2009) and plotted in polar coordinates (polar direction denoted the phase of the gait cycle—with angle θ that varies from 0 to 360° corresponding to 0 and 100% cycle, respectively—and radius denoted the mean EMG activity of the muscle). The CoA of the EMG waveform was calculated as the angle of the vector (first trigonometric moment) which points to the CoM of that circular distribution using the following formulas:

(1) $$A=\sum_{t=1}^{200}\left(\cos\theta_{t}\times EMG_{t}\right)$$

(2) $$B=\sum_{t=1}^{200}\left(\sin\theta_{t}\times EMG_{t}\right)$$

(3) $$CoA=\tan^{-1}(B/A)$$

The CoA has been used previously to characterize the overall temporal shifts of EMG or motoneuron activity (Yakovenko et al., 2002; Ivanenko et al., 2006; Sylos-Labini et al., 2011) and was chosen because it was impractical to reliably identify a single peak of activity in the majority of muscles. It can be helpful to understand if the distribution of muscular activity remains unaltered across different conditions.

### Statistics

Descriptive statistics included means ± standard deviation (SD) of the mean. The mean torque and peak-to-peak torque amplitudes were computed and compared across conditions. A repeated measure (RM) ANOVA was used to evaluate the effect of condition (on all parameters except for CoA) in healthy individuals. *Post-hoc* tests and multiple comparisons analysis were performed by means of the Bonferroni test. Circular statistics on directional data (Batschelet, 1981) were used to characterize the mean CoA for each muscle (see preceding text) and its variability across strides (angular SD). The Watson-William test was used for circular data (CoA) to evaluate the effect of condition in healthy individuals. Unpaired *t*-test was used to test differences in the exoskeleton torques and mean EMGs between controls and SCI patients. Statistics on Pearson's correlation coefficients was performed on the normally distributed, Z-transformed values. Reported results are considered significant for *p* < 0.05.

## Results

Results were presented and briefly discussed in the following manner to make clear comparison: first, comparisons on kinematic and kinetic data were made between EXO-assisted and EXO-unassisted walking conditions in healthy individuals; second, for the same two walking conditions, EMG data in lower limbs were presented; third, EMG data in EXO-assisted and NM slow walking were given; and finally, for the EXO-assisted walking condition, comparisons were made between healthy and SCI subjects based on the kinematic/kinetic and EMG measurements.

### EXO-assisted vs. EXO-unassisted: kinematics and kinetics in healthy subjects

**Figure 3:**
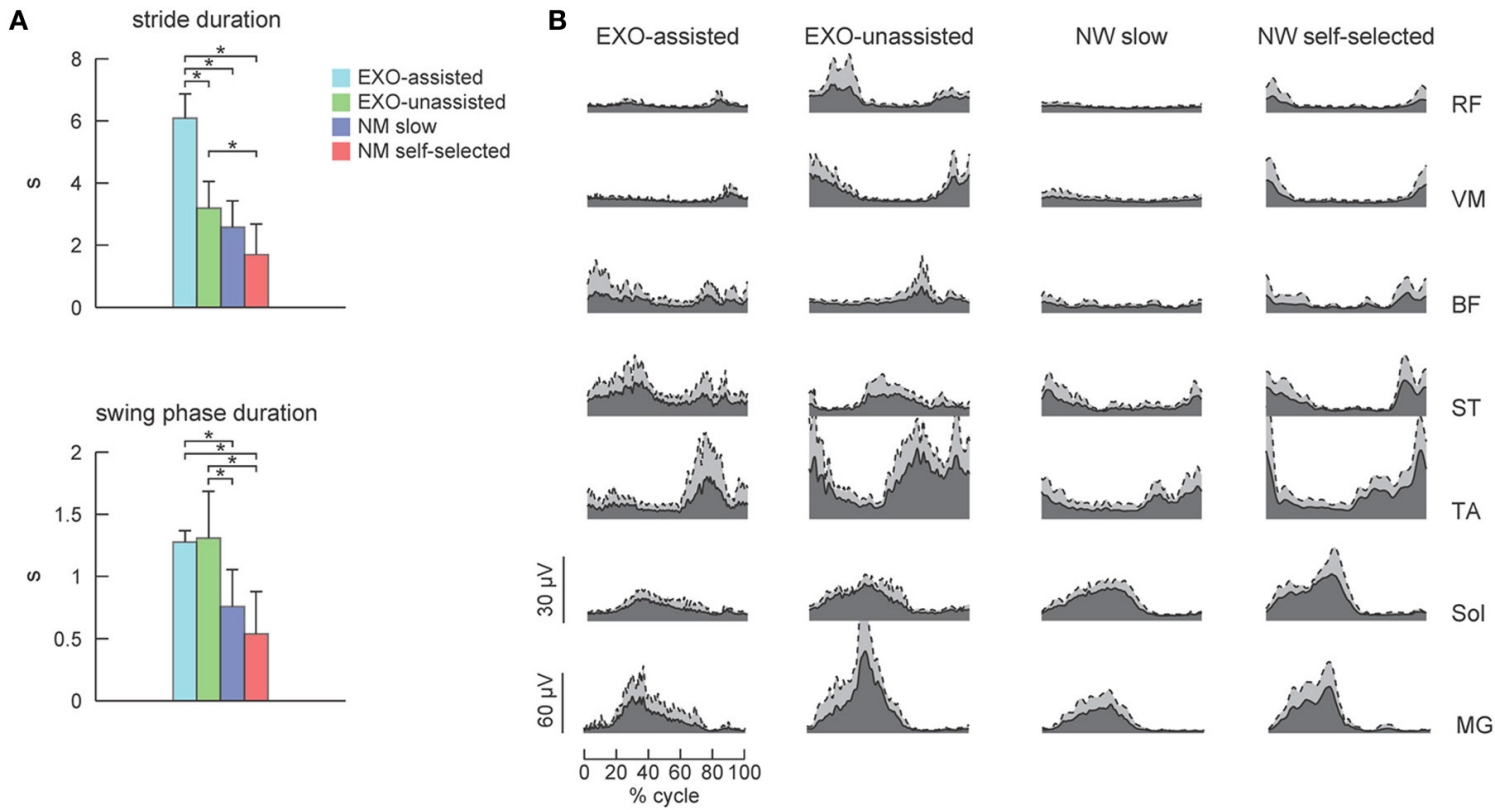
**EMG patterns in healthy subjects during walking in the exoskeleton and during normal overground walking. (A)** Stride and swing durations (mean +*SD*, *n* = 6) for each experimental condition. **(B)** Time course of ensemble-averaged EMG patterns (dark area, gray area corresponds to *SD*). Asterisks denote significant differences across conditions.

In general, as shown in Figure 3A, EXO-unassisted walking was faster than EXO-assisted walking (mean cycle duration was 3.2 ± 0.8 s vs. 6.1 ± 0.8 s). The swing phase durations were similar and the difference were caused by the dead time in stance, since in EXO-assisted walking, the subjects needed to move their trunk and trigger the swing step by step.

Figures 2A,B illustrates ensemble-averaged angular movements and joint torques in healthy subjects in these two walking conditions. The amplitude of the knee and hip joint angular movements in the sagittal plane were similar (Figure 2A), however, hip abduction was larger during assisted walking since lateral trunk movements were necessary to trigger the swing phase.

EXO-assisted walking in the exoskeleton requires relatively large torques in the hip and knee joints (Figure 2B). For instance, peak-to-peak amplitudes in the knee and hip joints (normalized to the wearer-EXO's weight) were about 1 Nm/kg (Figure 2C). Nevertheless, the torques that the exoskeleton applied to the subject (in the sagittal plane) were compatible to those exerted by subjects during normal overground walking (Winter, 1991). Note though that these torques were exerted by the exoskeleton (in order to move the subject's limbs and body) rather than by the subjects themselves. As expected, during unassisted walking these torques were very small (Figure 2B, right panel), which was dictated by the closed-loop torque control performance of the exoskeleton.

### EXO-assisted vs. EXO-unassisted: EMG patterns in healthy subjects

**Figure 4:**
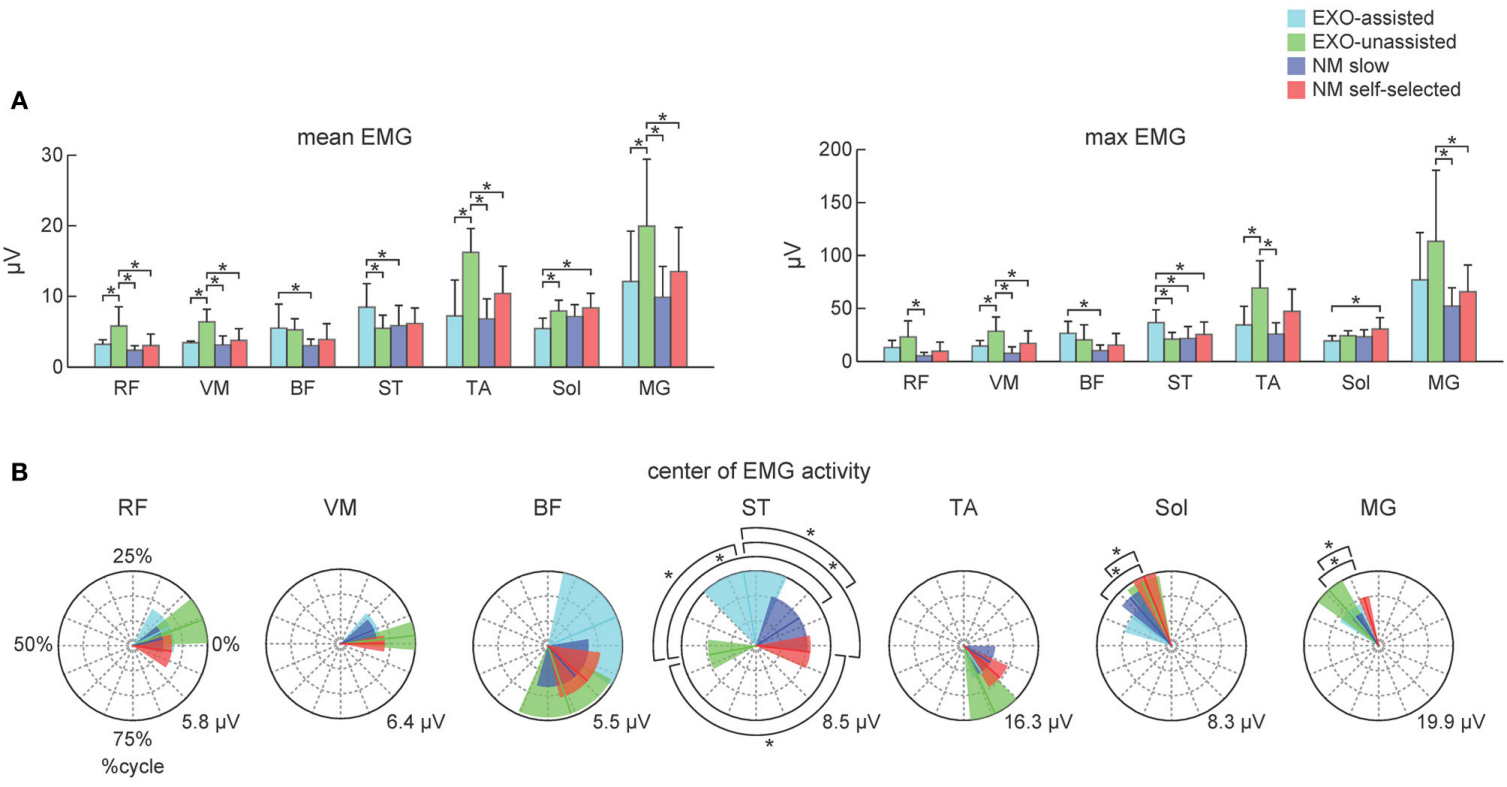
**Characteristics of EMG activity during assisted and normal walking in control subjects. (A)** Mean and maximum EMG activities (left and right panels, respectively) for each muscle (mean +*SD*). **(B)** Polar plots of the center of EMG activity. Polar direction denotes the relative time over the gait cycle (time progresses clockwise), radius of the vector denotes the mean EMG activity of the muscle and the width of the sector denotes angular *SD* (across subjects). Polar grid with circles was also shown to ease comparisons (the number in the right corner of each plot corresponds to the value of the external circle). Asterisks denote significant differences across conditions.

Figure 3B illustrates ensemble-averaged EMG patterns of leg muscles in control subjects in different walking conditions. During EXO-assisted walking the exoskeleton provided all necessary torques to support the body and move the legs forward while during unassisted walking the subjects moved their and exoskeleton's legs together. Accordingly, it was not surprising that during unassisted walking the amplitude of EMG activity was typically larger than that during assisted walking (Figure 3B, left two panels). Specifically, the mean activity was significantly larger for the RF, VM, TA, MG, and Sol muscles while it was comparable for BF and smaller for ST (Figure 4A). It is also worth noting that EMG waveforms differed for BF and ST: in particular, there was no activity in these muscles at the beginning of the stance phase during not-assisted walking (Figure 3B). The correlation analysis confirmed similarities in the EMG waveforms for RF, VM, MG, and Sol muscles and differences for BF, ST, and TA muscles between these two conditions (Table 2).

**Table 2:** **Pearson correlation coefficients (mean ±*SD*, *n* = 6) between EMG waveforms for different conditions in control subjects**.

**Muscle**	**Condition**	**EXO-assisted**	**EXO-unassisted**	**NW slow**	**NW self-selected**
RF	EXO-assisted	–	0.32 ± 0.29^*^	0.10 ± 0.21	0.07 ± 0.10
	EXO-unassisted	0.32 ± 0.29^*^	–	0.58 ± 0.52^*^	0.35 ± 0.27^*^
	NW slow	0.10 ± 0.21	0.58 ± 0.52^*^	–	0.32 ± 0.13^*^
	NW self-selected	0.07 ± 0.10	0.35 ± 0.27^*^	0.32 ± 0.13^*^	–
VM	EXO-assisted	–	0.70 ± 0.35^*^	0.41 ± 0.26^*^	0.29 ± 0.13^*^
	EXO-unassisted	0.70 ± 0.35^*^	–	0.61 ± 0.28^*^	0.56 ± 0.20^*^
	NW slow	0.41 ± 0.26^*^	0.61 ± 0.28^*^	–	0.50 ± 0.24^*^
	NW self-selected	0.29 ± 0.13^*^	0.56 ± 0.20^*^	0.50 ± 0.24^*^	–
BF	EXO-assisted	–	0.01 ± 0.34	−0.11 ± 0.29	0.25 ± 0.41
	EXO-unassisted	0.01 ± 0.34	–	0.26 ± 0.34	0.47 ± 0.51
	NW slow	−0.11 ± 0.29	0.26 ± 0.34	–	0.29 ± 0.09^*^
	NW self-selected	0.25 ± 0.41	0.47 ± 0.51	0.29 ± 0.09^*^	–
ST	EXO-assisted	–	−0.07 ± 0.33	0.20 ± 0.36	0.17 ± 0.17^*^
	EXO-unassisted	−0.07 ± 0.33	–	−0.24 ± 0.13^*^	−0.34 ± 0.10^*^
	NW slow	0.20 ± 0.36	−0.24 ± 0.13^*^	–	0.38 ± 0.13^*^
	NW self-selected	0.17 ± 0.17^*^	−0.34 ± 0.10^*^	0.38 ± 0.13^*^	–
TA	EXO-assisted	–	0.35 ± 0.49	0.14 ± 0.21	0.22 ± 0.07^*^
	EXO-unassisted	0.35 ± 0.49	–	0.33 ± 0.28^*^	0.26 ± 0.30
	NW slow	0.14 ± 0.21	0.33 ± 0.28^*^	–	0.72 ± 0.21^*^
	NW self-selected	0.22 ± 0.07^*^	0.26 ± 0.30	0.72 ± 0.21^*^	–
Sol	EXO-assisted	–	0.65 ± 0.34^*^	0.60 ± 0.30^*^	0.73 ± 0.34^*^
	EXO-unassisted	0.65 ± 0.34^*^	–	0.74 ± 0.14^*^	0.78 ± 0.25^*^
	NW slow	0.60 ± 0.30^*^	0.74 ± 0.14^*^	–	0.74 ± 0.14^*^
	NW self-selected	0.73 ± 0.34^*^	0.78 ± 0.25^*^	0.74 ± 0.14^*^	–
MG	EXO-assisted	–	0.67 ± 0.37^*^	0.65 ± 0.25^*^	0.74 ± 0.37^*^
	EXO-unassisted	0.67 ± 0.37^*^	–	0.72 ± 0.24^*^	0.87 ± 0.38^*^
	NW slow	0.65 ± 0.25^*^	0.72 ± 0.24^*^	–	0.79 ± 0.25^*^
	NW self-selected	0.74 ± 0.37^*^	0.87 ± 0.38^*^	0.79 ± 0.25^*^	–

Asterisks denote correlation coefficients significantly different from zero, t-test.

### EXO-assisted vs. NM slow walking: EMG patterns in healthy subjects

During normal walking, muscle activity typically increases with increasing walking speed (Ivanenko et al., 2006). Therefore, comparisons of normal and pathological gait are typically performed at similar walking speeds. Assisted walking in the exoskeleton (EXO assisted) was relatively slow compared to normal walking (Figure 3A). The swing phase duration was also longer for the EXO assisted condition (Figure 3A).

Since the movements of the limbs were performed by the exoskeleton, one would expect substantially lower muscle activity during assisted walking. Interestingly, contrary to our expectations, assisted walking in the exoskeleton was not accompanied by reduced EMG activity. In fact, the activity of most muscles (RF, VM, TA, MG, Sol) did not change significantly, while the activity of BF and ST even increased during assisted walking despite the slower walking speed in this condition (Figure 4A).

EMG waveforms of some leg muscles also differed between assisted gait and normal walking (Figure 3B). For instance, RF and VM activity contained additional bursts during the swing phase (Figure 3B), BF and ST muscles were activated in midstance and early swing during assisted walking (Figure 3B) and the center of activity of the ST muscle differed significantly between normal and assisted walking (Figure 4B). The correlation analysis showed low correlations for most muscles: only VM, Sol, and MG muscles demonstrated significant correlations between these two conditions (Table 2).

### SCI vs. healthy subjects in EXO-assisted condition: kinematics, kinetics, and EMG patterns

**Figure 5:**
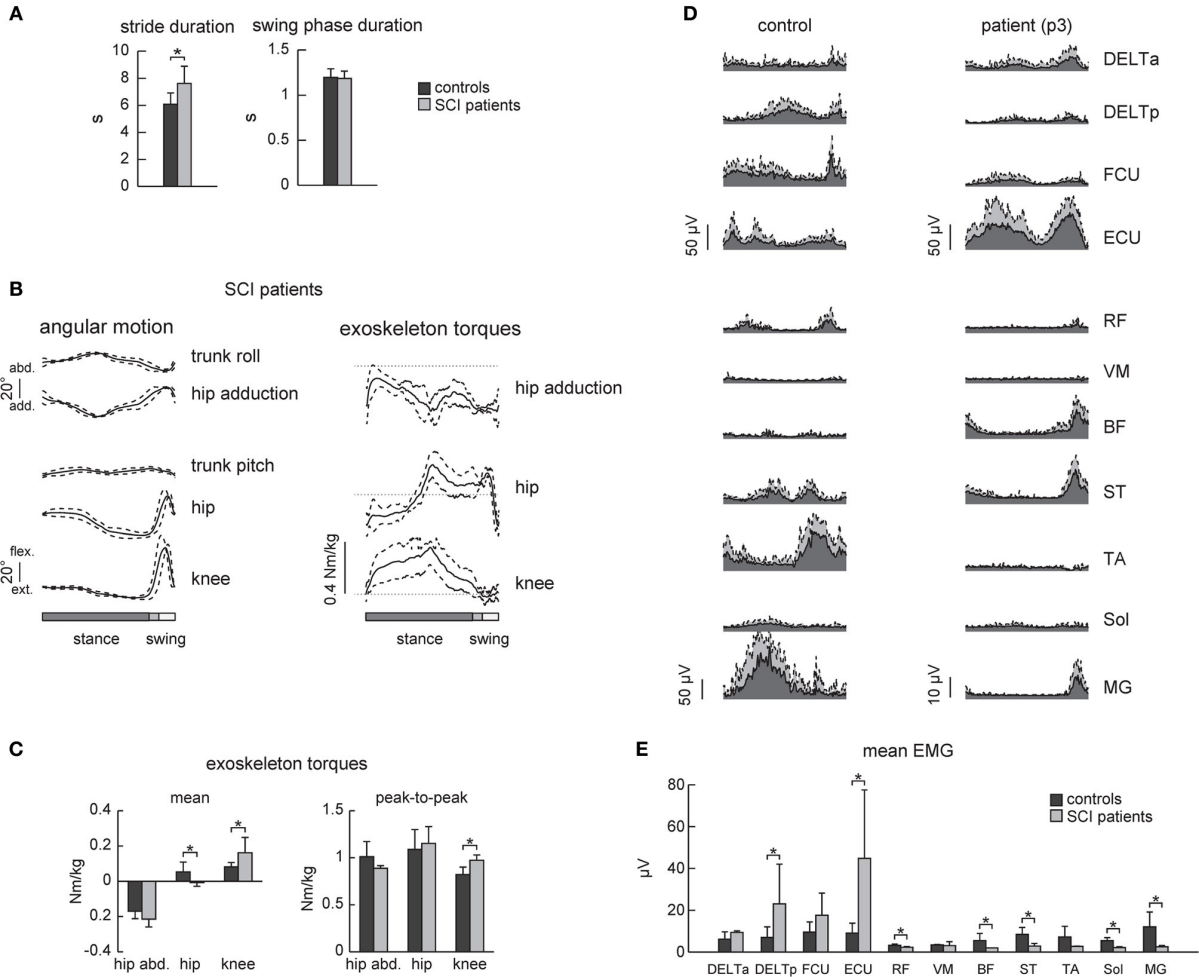
**EMG activity in healthy subjects and SCI patients during walking in the exoskeleton (“EXO-assisted” mode). (A)** Joint angles and exoskeleton torques recorded in SCI patients during walking in the exoskeleton. The same format as in Figure 2. **(B)** Mean torques and peak-to-peak oscillations of torques in control subjects and patients. **(C)** An example of EMG activity in the upper and lower limb muscles in a healthy subject (left) and SCI patient (right, p3 Table 1) during walking in the exoskeleton. Note EMG activity in the upper limb muscles in both subjects. Note also some EMG activity in the ST, BF, and MG muscles in the patient despite neurologically complete paraplegia. **(D)** Mean (+ *SD*) EMG activity of the upper and lower limb muscles. Asterisks denote significant differences. **(E)** Mean EMG activities for each muscle (mean + *SD*).

SCI patients walked slightly slower than the control subjects (Figure 5A, on average, the cycle duration was 6.1 ± 0.8 s in control subjects and 7.6 ± 1.1 s in SCI patients), though the swing phase duration (Figure 5A right panel) and the angular movements (Figure 5B left panel) were similar. The amplitude (peak-to-peak) of the exoskeleton torques was also similar (only the knee torque was larger, Figure 5C), suggesting that the exoskeleton provided the main forces for stepping in both control and SCI subjects.

Despite similarities in the kinematics and dynamics of movements, EMG patterns differed in SCI patients. Overall, they used more upper limb muscles for stepping (DELTp and ECU Figure 5E) though there was also variability in using the arms muscles between subjects (compare, for instance, the control and the SCI subject in Figure 5D). EMG activity in the lower limb muscles was typically minute if any in SCI patients, though one SCI patient demonstrated consistent activity in the BF, ST, RF, and MG muscles during the swing phase and beginning of stance (Figure 5D right panel).

## Discussion

We investigated the effect of walking with an exoskeleton on the muscular activation patterns in healthy subjects and SCI patients. Strikingly, despite exoskeleton assistance in both posture and leg movements, the overall muscle activity level in healthy subjects was not reduced at all, as one would expect, further supporting the importance of sensory input and suitability of using robotic exoskeletons for entraining lumbosacral locomotor circuitry. The results also showed a non-linear reorganization of EMG patterns under different walking conditions (Figures 3–5, Table 2). Below we discuss the findings in the context of adaptability of locomotor patterns and human-machine interactions.

### EMG patterns in healthy subjects

To assess similarities in the EMG waveforms across conditions, we used both the correlation analysis and calculated the center of EMG activity in the gait cycle. Both invariant features and significant variations in the EMG waveforms were observed across different walking conditions (Figure 4, Table 2).

For instance, the correlation analysis revealed that in EXO-assisted and normal slow walking Sol and MG muscles demonstrated significant correlations (Table 2), which could be explained by the fact that the exoskeleton ankles were not powered and in both conditions human ankles were actively contributing to locomotion and to antigravity calf muscle activity during foot loading in the stance phase (Nielsen and Sinkjaer, 2002). The most variable pattern was generally observed in the hamstring muscles (BF and ST). This can be explained by the important contribution of stretch reflexes in this muscle in the context of a “passive” contribution (Duysens et al., 1998), but it can also be interpreted in terms of the more proximal muscles being less dependent on sensory feedback than the distal ones (in the context of “active” contribution from central sources). Another explanation can be related to the fact that the hamstring muscle (in particular, the semimembranosus and semitendinosus muscles) is specifically involved in the locking of the erect posture by producing a tonic activation against the action of gravity (Cheron et al., 1997). Indeed, an anticipated inhibition of the hamstring activity worked in conjunction with the phasic activation of the TA and the action of gravity (Cheron et al., 1997). In the present “EXO-assisted” situation, such inhibitory modulation related to normal graviception can be disturbed by the presence of these artificial forces. The context-specific function of the hamstring muscle was also reported in other experimental conditions (Ivanenko et al., 2000; Sylos-Labini et al., 2014).

The amplitude of EMG activity varied across conditions. Walking in EXO-unassisted mode in healthy individuals was accompanied by the augmented motor output (Figures 3, 4), likely due to additional inertia and weight of the exoskeleton. Strikingly, however, walking in the EXO-assisted mode was not accompanied by the reduction of leg muscle EMG activity despite limb movement assistance. This can be explained in part by the important contribution of afferent feedback to the pre-programmed motoneuronal drive (Nielsen and Sinkjaer, 2002), different biomechanical demands and the “active” nature of stepping in the exoskeleton (the subject was not fully “relaxed,” needed to maintain the upper trunk posture and provide small lateral trunk displacements to trigger step transitions) even though the limb movements were guided by the exoskeleton. Another possible cause could be the intermittent contact between the exoskeleton and the subject. The brace connections were not tight and had slag (for comforts and to prevent overloading human joints since minor misalignments could not be avoided.). It could be that the subject was ambulating on his own and the exoskeleton was acting as disturbances to the subject due to the intermittent contacts.

Adaptive non-linear changes in both amplitude and temporal envelope have been reported in other walking conditions as well (Hidler and Wall, 2005; Israel et al., 2006; Lam et al., 2008; Van Asseldonk et al., 2008; Moreno et al., 2013). For instance, with body weight unloading (Ivanenko et al., 2002), most muscles (e.g., gluteus maximus and distal leg extensors) decrease their activity, while other muscles demonstrate a “paradoxical” increment of activation (e.g., quadriceps) or considerable changes in the activation waveforms (hamstring muscles). Even the amplitude of EMG activity of “anatomical” synergists may diverge remarkably: lateral and medial gastrocnemius muscles at different walking speeds (Huang and Ferris, 2012), soleus and gastrocnemius muscles at different levels of limb loading (McGowan et al., 2010). In addition, muscle activity patterns are shaped by the direction of progression (e.g., forward vs. backward, Grasso et al., 1998, or walking along a curved path, Courtine et al., 2006). In particular, such studies suggest that a comparison of normal and pathological gait should be preferably performed in the same stepping conditions.

Taken together, the data support the idea of plasticity and distributed networks for controlling human locomotion (Scivoletto et al., 2007; Ivanenko et al., 2013). Tens of muscles participate in the control of limb and body movements during locomotion, and redundancy in the neuromuscular system is an essential element of gait adaptability (Winter, 1989; Cai et al., 2006; Noble and Prentice, 2006; Molinari, 2009; Duysens et al., 2013; Ivanenko et al., 2013). Due to muscle redundancy, various neuromotor strategies may exist to compensate for decreased muscle strength and pathology (Grasso et al., 2004; Goldberg and Neptune, 2007; Huang and Ferris, 2012; Gordon et al., 2013).

### EMG patterns in SCI patients

Flexibility and adaptability of locomotor patterns are evident from monitoring and analyzing the spatiotemporal spinal segmental output after SCI (Grasso et al., 2004; Scivoletto et al., 2007). For instance, in motor incomplete paraplegics who recovered independent control of their limbs, an additional activation burst, related to abnormal activation of the quadriceps muscle, is often present in the lumbosacral enlargement (Ivanenko et al., 2013). Patients can be trained to step with body weight support unassisted, but they use activity patterns in individual muscles that were often different from healthy individuals (Grasso et al., 2004).

In this study we used the reference patterns based on pre-recorded trajectories from unimpaired volunteer walking in the device while it is operated in a transparent mode. Other approaches may be based on patient specific patterns by recording the gait trajectory while the patient walks with manual assistance (Aoyagi et al., 2007), but this may be done only in individuals with less severe paresis of the lower limbs. Further investigations are needed regarding the possible effect that the selected reference gait pattern may have on the findings and also regarding possible solutions for reference gait pattern customization for SCI.

Patients with severe SCI disorders frequently show EMG patterns different from those of healthy individuals suggesting that human spinal cord can interpret differently loading- or velocity-dependent sensory input during stepping (Beres-Jones and Harkema, 2004). Complete paraplegics also use more their arms and largely rely on proximal and axial muscles to assist the leg movements and balance control (Figures 5D,E, see also Grasso et al., 2004). During assisted walking in the exoskeleton, complete paraplegics typically showed little if any leg muscle activity (Figure 5E). Only one patient (p3, Table 1) demonstrated consistent activity in the BF, ST, RF, and MG muscles during swing and beginning of stance (Figure 5D right panel), suggesting the contribution of stretch- or loading-related afferent inputs to muscle activity (Maegele et al., 2002; Beres-Jones and Harkema, 2004; Grasso et al., 2004). Nevertheless, this reflex-related activity might be beneficial for potential gait rehabilitation since there is a relationship between facilitation of segmental reflexes and the ability to recover gait (Dietz et al., 2009; Thompson and Wolpaw, 2014). Thus, in addition to gait assistive aspects of exoskeleton robotic devices in severely paralyzed individuals, the proposed approach may also be beneficial for gait rehabilitation. We did not test in this study the effect of robot-assisted gait training in persons with SCI. Longer sessions would be required to evaluate the adequate learning paradigm, likely in combination with other central pattern generator-modulating therapies (Roy et al., 2012; Guertin, 2014) and biofeedback that might help the patients to adapt their movement patterns and to improve their motivation (Lünenburger et al., 2007).

### Conclusions

Overall, the results are consistent with a non-linear reorganization of the locomotor output when using the robotic stepping devices. The findings may contribute to our understanding of human-machine interactions and adaptation of locomotor activity patterns. Locomotor movements can be accommodated to various external conditions, and some of the suggestions in this article may possibly be revised as empirical data on the sensorimotor interactions when walking with different types of exoskeletons accumulate. The effect of learning and adaptation is also an interesting avenue of future research. Such investigations may have important implications related to the construction of gait rehabilitation technology.

### Conflict of interest statement

The authors declare that the research was conducted in the absence of any commercial or financial relationships that could be construed as a potential conflict of interest.
